# Evaluation of Two Fully Automated Setups for Mycotoxin Analysis Based on Online Extraction-Liquid Chromatography–Tandem Mass Spectrometry

**DOI:** 10.3390/molecules25122756

**Published:** 2020-06-15

**Authors:** Edvaldo Vasconcelos Soares Maciel, Karen Mejía-Carmona, Fernando Mauro Lanças

**Affiliations:** São Carlos Institute of Chemistry, University of São Paulo, São Carlos, SP 13560-970, Brazil; edvaldovasconcelos@usp.br (E.V.S.M.); ksmejiac@gmail.com (K.M.-C.)

**Keywords:** mycotoxins, liquid chromatography, sample preparation, automation, miniaturization

## Abstract

Mycotoxins are secondary metabolites of fungi species widely known for their potentially toxic effects on human health. Considering their frequent presence in crops and their processed food, monitoring them on food-based matrices is now an important topic. Within such a context, the sample preparation step is usually mandatory before the chromatographic analysis, due to the complexity of matrices such as nuts, cereals, beverages, and others. For these reasons, we herein present the evaluation of two greener setups, based on the automation and miniaturization of the sample preparation step for mycotoxin analysis in different beverages. Firstly, we describe an analytical method based on a multidimensional assembly, coupling a lab-made microextraction column (508 µm i.d. × 100 mm) to a UPLC–MS/MS for the analysis of ochratoxin A in beverages. This configuration used a synthesized sorbent phase containing C18-functionalized graphene–silica particles, which exhibited excellent extraction performance, as well as being reusable and cheaper than commercially available extractive phases. Sequentially, a second setup, based on a multidimensional capillary LC coupled to MS/MS, was assessed for the same purpose. In this case, a graphene oxide-based capillary extraction column (254 µm i.d. × 200 mm) was used as the first dimension, while a C18 analytical capillary column performed the mycotoxin separation in beverages. Although this second one has similarities with the first, we focused mainly on the benefits related to the link between a miniaturized/automated sample preparation device with a capillary LC–MS/MS system, which made our analysis greener. Additionally, the chromatographic efficiency could even be enhanced.

## 1. Introduction

Mycotoxins are toxic secondary metabolites produced by some filamentous fungi and whose presence has been detected mainly in agricultural products and processed food [1,2]. A recent review published by Eskola et al. [3] estimated that more than 60% of all crops around the world contain mycotoxins, and several classes can simultaneously be found in food. Contamination can happen at any stage of cultivation, harvesting, processing, or storage, when environmental conditions are favorable for the proliferation of these fungi, especially humidity and temperature [3]. Chemical and environmental factors, such as water content, pH, available nutrients, substrate, and the physical integrity of the plant and grain, also affect the growth of fungi and the mycotoxins [4]. Food contamination by fungi is an important issue in food safety, since the compounds produced by these microorganisms have severe consequences for human and animal health [3]. Mycotoxins present in food are usually produced by fungi of the genera *Aspergillus*, *Fusarium*, and *Penicillium*. Among the most relevant groups, with the highest occurrence and health risks, are aflatoxins (aflatoxin B_1_, AFB_1_), ochratoxins (ochratoxin A, OTA), trichothecenes (deoxynivalenol, DON), fumonisins (fumonisin B_1_, FB_1_), and zearalenone (ZEA) [5]. The International Cancer Research Agency (IARC) classified the mycotoxins depending on their toxic effects: group 1 as proven carcinogen agents, group 2A as probable carcinogen agents, and group 2B as possible carcinogen agents to humans [6].

Aflatoxins are the most relevant and studied group, which comprises 20 different metabolites, among them: aflatoxins B_1_ (AFB_1_), B_2_ (AFB_2_), G_1_ (AFG_1_), G_2_ (AFG_2_), M_1_ (AFM1), and M_2_ (AFM_2_) [7]. In general, aflatoxins are extremely toxic, possessing carcinogenic metabolites (classified in group 1 by the IARC [6]) being also associated with hepatocellular carcinoma (HCC) or liver cancer. Aflatoxins are commonly reported in several types of cereals, nuts, and spices, as well as their metabolites (AFMs), which can be found in milk and dairy products [8,9]. Aflatoxins show a high resistance to sterilization, pasteurization, and thermal food treatments, being of the utmost importance for preventing food contamination, especially with AFB_1_ [7].

In the same way, ochratoxins A, B, and C are produced by several species of the *Aspergillus* genus in tropical climates, as well as by the *Penicillium* genus in temperate climates. Among them, ochratoxin A (OTA) is the most toxic and frequently reported in foodstuffs. OTA has been found in a wide variety of foods, the main sources being cereals, wine, and coffee [10,11]. Usually, this mycotoxin is stable at high temperatures and in acidic environments [12]. Their most notable adverse effect is nephrotoxicity, associated with Balkan endemic nephropathy (BEN), which causes tubular degeneration and interstitial fibrosis. Additionally, OTA is a teratogenic and carcinogenic agent, classified by IARC in group 2B [7].

Zearalenone (ZEA), a metabolite from the *Fusarium* genus, and is currently classified as a potential estrogenic agent, due to its ability to bind to estrogen receptors in animals, causing hormonal deregulations leading to atypical fetal development in mammals and infertility [13]. Other effects include hepatic damages and renal lesions in rodents, since they are often peeking in farm warehouses where cereals are stored and, also, a decrease in cows’ milk production due to its toxicity affecting the mammary glands [14]. Generally, ZEA can be found in rice, maize, nuts, and other foodstuffs, which show it to be a relevant chemical compound for analysis [14,15].

The analysis of mycotoxins can be done by qualitative methods such as thin-layer chromatography (TLC) and enzyme-linked immunosorbent assays (ELISA), or by quantitative ones, such as HPLC coupled to a fluorescence detector (FLD) or tandem mass spectrometry (MS/MS) [16]. The use of LC–MS/MS allows the unambiguous confirmation of mycotoxins and their metabolites by their mass spectra information. Nowadays, this is one of the most critical techniques that provides much information, allowing for the simultaneous detection of multi-present mycotoxins and emerging mycotoxins or masked metabolites [5]. However, before the instrumental analysis of mycotoxins can be carried out successfully, a previous sample preparation step is usually mandatory [17]. For this reason, mycotoxins are extracted from the food matrix and added to a more appropriate media or solvent [18]. After that, the extract is subjected to a cleaning step, usually employing liquid–liquid extraction (LLE) or solid-phase extraction (SPE) cartridges, preferably packaged with an immunoaffinity sorbent, which are known as immunoaffinity columns (IACs). IACs have been one of the most relevant achievements and the preferred method for mycotoxin analysis, being a highly selective sorbent due to the use of antibodies bound to a support material [17,19]. Among the advantages of the IAC employment in mycotoxin analysis are the attainment of clean extracts and low detection limits (0.1–0.2 ng g^−1^), selectivity, smooth operation, and less organic solvent consumption. However, it has some disadvantages, such as the low number of antibodies in the phase and the high dependence on the extraction conditions, such as the aqueous media, controlled pH, ionic strength, and extract concentration. Besides, IACs have a short half-life, as antibodies biodegrade and are incompatible with some organic solvents that denature them [19].

Although a satisfactory sample preparation can be obtained by conventional SPE and LLE techniques, they are often related to high levels of reagent consumption, waste generation, high cost, and sample/stationary phase requirements. Therefore, novel methods to analyze mycotoxins have emerged, focusing on the automation and miniaturization of the sample preparation procedure towards the development of greener approaches [20]. In this case, techniques such as online SPE [21,22,23,24,25] in-tube SPME (solid-phase microextraction) [26], turbulent flow chromatography (TFC) [17,27,28], and customized procedures derived from them [17,29,30,31], arose as suitable tools to perform such analyses while benefits emerging from automation/miniaturization could be reached. In this context, downsizing the sample preparation represents a promising way to be in accordance with green chemistry principles, as it is related to reduced consumption of chemicals/samples, and a reduced generation of toxic waste. Additionally, applying automation concepts helps to overcome the problems frequently related to the offline conventional sample preparation approaches, such as laborious and time-consuming procedures, the manual handling of samples, and in some cases, poor accuracy or precision [17]. Recent trends for mycotoxin analysis seem to prioritize the miniaturization and automation of the sample preparation approach, allowing them to obtain high-throughput analytical methods [20].

For these reasons, we herein present the evaluation of two multidimensional LC systems based on the automation and miniaturization of the sample preparation steps for mycotoxin analysis in different food matrices. First, an analytical method, based on the coupling of a lab-made microextraction column (508 µm i.d. × 100 mm) to a UPLC–MS/MS, was employed for the analysis of OTA in beverages. This approach uses a synthesized sorbent phase containing C18-functionalized graphene–silica particles. Sequentially, a modern setup using a multidimensional capillary LC coupled to MS/MS was assessed for the same purpose. In this case, a graphene oxide-based capillary column (254 µm i.d. × 200 mm) was used as the first dimension, while a C18 analytical capillary column performed the mycotoxin separation in beverages. Although both setups have similarities, we focused mainly on the benefits related to the link between a miniaturized/automated sample preparation device and a capillary LC–MS/MS system. This one made our mycotoxin analysis greener, whereas the chromatographic efficiency could have been enhanced. Ultimately, this work aims to gather the analytical results and prospects for these two fully automated and miniaturized setups, supporting them as greener alternatives specially developed to extract and analyze mycotoxins from complex samples such as wine, beer, and coffee.

## 2. Results and Discussion

### 2.1. Setup 1: Ochratoxin A Analysis by Packed Microextraction Column Online Coupled to UPLC–MS/MS

#### 2.1.1. Chromatographic Conditions for the Analysis of Ochratoxin A

**Figure 1:**
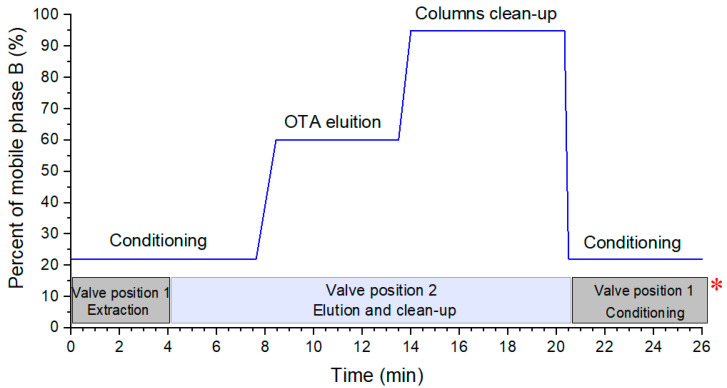
Step elution gradient for the analysis of OTA (Ochratoxin A) by an extraction microcolumn coupled to UPLC–MS/MS, indicating the mobile phase composition in the UPLC pumps, A: H_2_O:0.1% formic acid and B: ACN (Acetonitrile): 0.1% formic acid (blue line). The box below the gradient indicates the configuration of the switching valve (*).

Initially, the microextraction column was online-coupled to the UPLC–MS/MS, using both loading and mobile phases acidified with 0.1% of formic acid to keep the OTA protonated and favoring its ionization. For the OTA analysis, a step-gradient elution program (Figure 1, see blue line) was used, which allowed the performance of the following steps: (1) the conditioning of the analytical column and extraction of OTA in the microcolumn, (2) the elution and determination of OTA, (3) clean-up, and (4) the conditioning of both columns. It is noteworthy that the selected gradients were optimized to reduce matrix interferents, to obtain the highest OTA analytical signal, and to avoid the carryover effects.

As previously observed by our research group, a mobile phase of H_2_O:ACN (acetonitrile) 78:22 *v*/*v* was used from 0 to 7.63 min for conditioning the analytical column, as well as for loading the sample into the microextraction column [26]. In the second isocratic step, from 8.5 to 13.5 min (OTA elution), three ACN compositions (50%, 60%, and 70%) were tested. OTA’s largest peak area was obtained with 60% ACN with an elution time of 11.2 min. Later, the clean-up step was verified by injecting an OTA standard solution followed by a blank of water. In this way, 95% of ACN for 6.30 min was enough to clean the analytical system. Finally, the chromatographic method returned to the initial gradient step for conditioning both columns for the next injection.

#### 2.1.2. Extraction Valve Programming Events

The switching valve time events were programmed to determine the minimum extraction time and the percentage of ACN in the loading phase to retain the OTA in the microextraction column (see boxes in Figure 1). In these experiments, the loading phase (H_2_O: ACN 78:22, *v*/*v*) flow rate was 0.100 mL min^−1^. OTA standard solutions (20.0 µg L^−1^) were consecutively injected, varying the time values from 6 to 1 min, aiming to determine the suitable loading time. These results (Figure S1) showed that by using a loading time from 1 to 4 min, the OTA area does not decrease significantly, unlike from 4 to 6 min, where a progressive reduction in the chromatographic peak was observed. For these reasons, 4 min was chosen as the loading time, because for more complex matrices, such as coffee, the use of longer loading times helped to eliminate more interfering analytes, avoiding the general contamination of the analytical system.

Sequentially, the loading phase composition was evaluated at four different levels: 5%, 10%, 15%, and 22% of ACN (flow rate, 0.100 mL min^−1^; loading time, 4 min). The OTA peak areas obtained by using 5%, 10%, 15%, and 22% of ACN were: 9.91 × 10^4^, 9.50 × 10^4^, 9.29 × 10^4^, and 7.98 × 10^4^, respectively. Therefore, as the OTA area was not significantly affected by using 5% to 15% of ACN, the last was chosen as the loading phase composition, since fewer interferent signals were observed at this value.

Finally, the loading flow was evaluated at three levels: 0.100, 0.150, and 0.200 mL min^−1^. The higher OTA response was achieved at a flow rate of 0.150 mL min^−1^, possibly because at the lower flow of 0.100 mL min^−1^, a satisfactory sorption interaction between the OTA and the sorbent phase was not provided, while at a higher flow of 0.200 mL min^−1^, part of the OTA stayed in the mobile phase, going to waste. Thus, 0.150 mL min^−1^ was fixed as the optimal value.

Shortly, after all the tests, the optimal value for each parameter was: loading phase of H_2_O:ACN (85:15, *v*/*v*), 4 min of loading time, and 0.150 mL/min loading flow rate. Within such a context, injections of instant coffee and wine spiked with OTA at 2.0 µg L^−1^ were performed to verify the system performance, and will be discussed in the following section.

#### 2.1.3. Applicability of the Extraction Microcolumn Coupled to UPLC–MS/MS for the Analysis of OTA in Beverages

**Figure 2:**
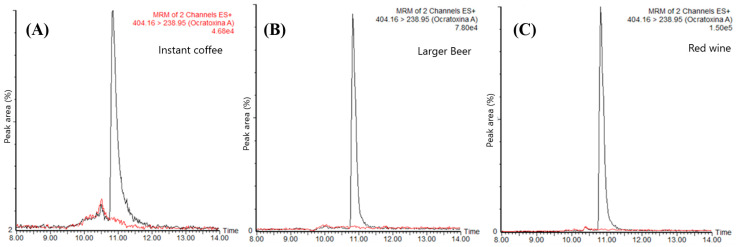
Superposition of the SRM (selected reaction monitoring) chromatograms of OTA ion transition (*m*/*z* 404.1 → *m*/*z* 238.9) from the analysis of (**A**) instant coffee, (**B**) beer, and (**C**) red wine samples fortified with OTA at 2.0 μg L^−1^ (black trace) and blank matrix sample (red trace).

The proposed method was evaluated with samples of instant coffee, beer, and red wine, with OTA spiked at 2 µg L^−1^ to verify its applicability to them and the OTA response in each one. Table 1 shows the comparison of the signal-to-noise (S/N) ratio obtained from the different matrices at the same concentration, and the corresponding chromatograms are shown in Figure 2. The SRM (selected reaction monitoring) chromatograms (Figure 2), corresponding to the OTA’s highly selective MS/MS transition, show that the method can be applied to OTA determination in instant coffee, beer, and wine. The OTA retention time signal was observed at 11.2 min, without any interferent peak being detected. The analysis of wine and beer was more straightforward than for coffee, as these samples were already in the liquid state, requiring just degassing and filtering before injection into the system. This reduced the sample preparation time compared to instant coffee, which needed to be dissolved in hot water, centrifuged, and filtered before the injection.

A higher OTA response was obtained in beer and wine samples within such a context as in coffee since at the same spiked concentration, a higher S/N ratio was obtained in these two matrices (Table 1). For this reason, lower limits of quantification were achieved in beer (malt: 74 ng L^−1^, lager: 86 ng L^−1^) and wine (68 ng L^−1^) when compared to instant coffee (351 ng L^−1^). These values were determined considering a signal-to-noise ratio of 10:1 for each matrix. From the analyzed matrices, it can be inferred that the instant coffee sample was the most complex one, presenting more significant suppression of the OTA signal in the MS/MS detector (matrix effect). During the analyses, it was observed that the OTA response varied depending on the type of beer; a similar behavior was observed for different brands of instant coffee samples. This observed behavior suggests that the matrix effects (ME) should be carefully evaluated for each matrix before the routine use of the method to analyze OTA.

#### 2.1.4. Method Overview

The proposed method, based on the first setup, was successfully implemented with potential applications in the analysis of OTA in wine, beer, and instant coffee. The employment of a non-selective sorbent for the OTA extraction proved to be advantageous, as this sorbent phase showed an excellent affinity for it, providing good retention with the initial extraction conditions evaluated. Additionally, it provided a good sensitivity for the OTA determination in beer, wine, and instant coffee samples, at spiked levels of 2 μg L^−1^, and their determination below the legally allowed concentration levels of OTA. Other advantages of this microextraction column were the reduced quantity of sorbent employed (20 mg) and its robustness, being re-used over 250 times without losing its original sorption characteristics. This sorbent was simple to synthesize from inexpensive raw materials (Mejía-Carmona et al. [32]) and easier to use compared to the immunoaffinity ones, which require special attention on the solvent composition and the use of buffer solutions [19]. Additionally, the high usability of this microextraction column shows the material’s excellent stability and a possible reduction in the method costs.

Moreover, the full automation of the analytical method brings several perceived advantages. Among them, it should be highlighted that the excellent extraction was performed with suitable LOQs and the achievement of an effective clean-up, and the complete analysis was performed in a single analytical run with a considerable reduction in the sample preparation steps. As an example, for the wine and beer analysis, the samples were just degassed and filtrated before their direct injection into the discussed analytical system, thus reducing the sample handling and possible contamination. Besides, in this system, the solvent consumption and waste generation were minimized by operating both columns (extraction and analytical) at lower flow rates (<0.200 mL min^−1^) than those utilized in most conventional systems.

### 2.2. Setup 2: Aflatoxins, Ochratoxin A, and Zearalenone Analysis by Multidimensional Capillary LC–MS/MS

#### 2.2.1. Chromatographic Separation

**Figure 3:**
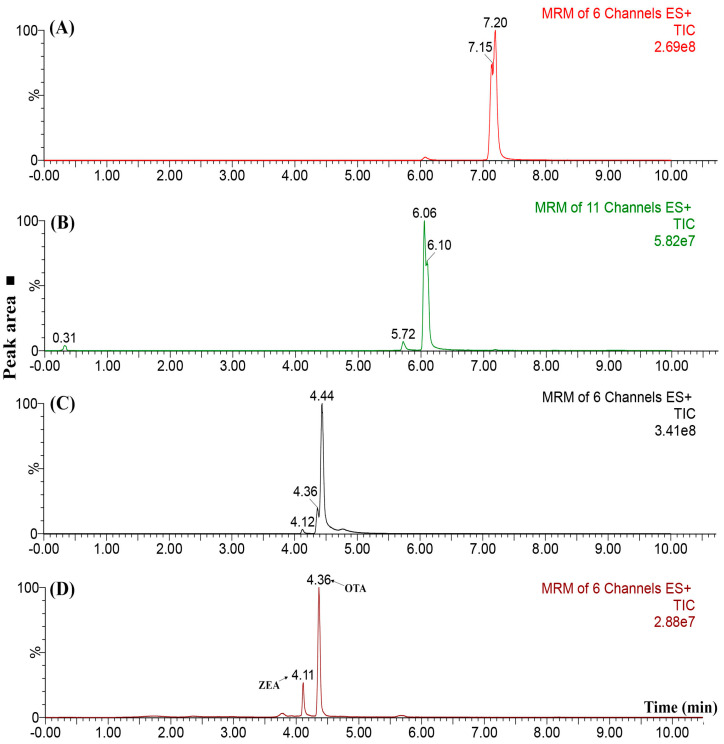
Chromatograms of a mixture containing OTA and ZEA (zearalenone) (15 µg L^−1^) aiming to achieve a satisfactory liquid chromatography separation. (**A**) Isocratic elution employing ACN:H_2_O (30:70, *v*/*v*); (**B**) acidification with 0.1% formic acid into mobile phases and the employment of an elution gradient; (**C**) enhancing the flow rate from 8 to 10 µL min^−1^ and (**D**) enhanced condition by fixing the flow rate at 10 µL min^−1^ and performing a less-steeped elution gradient.

Firstly, the liquid chromatographic separation between our two initial compounds (OTA and ZEA) was investigated by injections of a standard solution, containing both analytes, at a concentration level of 15 µg L^−1^. Figure 3 depicts the obtained results (from A to D), through which the improvements obtained in the resolution, analysis time, as well as a reduction in peak band broadening, can be seen. In this case, the progress is attributed to the chromatographic parameters that were modified between these injections. The trace (A) in Figure 3 was conducted in isocratic mode using a mobile phase composition of ACN:H_2_O (30:70, *v*/*v*), without being acidified. This isocratic elution mode can explain the higher elution time observed in this case. As it is known, electrospray ionization (ESI, in this case operating in positive mode) can be highly influenced by the pH or the presence of matrix interferents. Thus, the weak signal observed for ZEA (a small peak near 6 min) could be improved by decreasing the pH and favoring ionization, as observed in Figure 3B, where the ZEA signal was enhanced by the addition of 0.1% formic acid on both mobile phases. Additionally, the reduction in the retention times could be attributed to the use of the elution gradient instead of isocratic mode. The following step consisted of improving the resolution between the peaks, as well as fixing the subtle break on top of the OTA peak (Figure 3B,C). For this purpose, the mobile phase flow rate increased from 8 µL min^−1^ to 10 µL min^−1^ to diminish the peak width, while an elution gradient that was steeped less was applied to maintain the separation between the ZEA and OTA. Finally, the chromatogram showed in (D) was achieved by keeping the mobile phase flow rate at 10 µL min^−1^. Additionally, the formic acid percentage was increased to 0.5%, and the elution gradient was slightly modified by enhancing the ACN percentage, while decreasing the gradient steepness curve (Figure S3, Supplementary Materials).

#### 2.2.2. The Fully Automated Extraction Procedure

After that, with an excellent chromatographic separation already achieved, the other part of the multidimensional system was assessed. In this step, the main goal was to find an ideal condition for the sorption of ZEA and OTA into the capillary extraction column. Figure S4 (Supplementary Materials) shows the variation of the parameters’ loading phase and loading time, commonly considered in automated sorption-based extraction procedures. As can be seen, the higher loading time (1 min) reported the best response for the total peak area of each analyte, which suggests a good sorption interaction between the target compounds and the GO-Sil extractive phase. As a result, 1 min was fixed as the loading time for the subsequent analysis. Moreover, the loading flow employed was evaluated in three different values when the sample was pumped to the extraction column: 10, 25, and 50 µL min^−1^. Figure S4 (Supplementary Materials) depicts the obtained results for each analyte’s chromatographic peak area, indicating the intermediate value of 25 µL min^−1^ as the best among them. This trend indicates that a loading flow of 10 µL min^−1^ was probably insufficient to provide a satisfactory sorption interaction between the mycotoxins and the extractive phase. In contrast, 50 µL min^−1^ seemed to be too high, causing analytes to be more diluted when pumped inside the extraction column, which compromised the extraction performance. Additionally, for the loading solvent, an aqueous solution containing 22% ACN was chosen, taking into consideration previous experience from our research group [26].

#### 2.2.3. Applicability of the Proposed Analytical Method in Wine Samples

After establishing the best analytical condition in system 2, three wine samples were tested for the presence of OTA and ZEA. These analyses were performed to evaluate the performance of the fully automated and miniaturized method in real samples at concentration levels usually considered in the literature.

**Figure 4:**
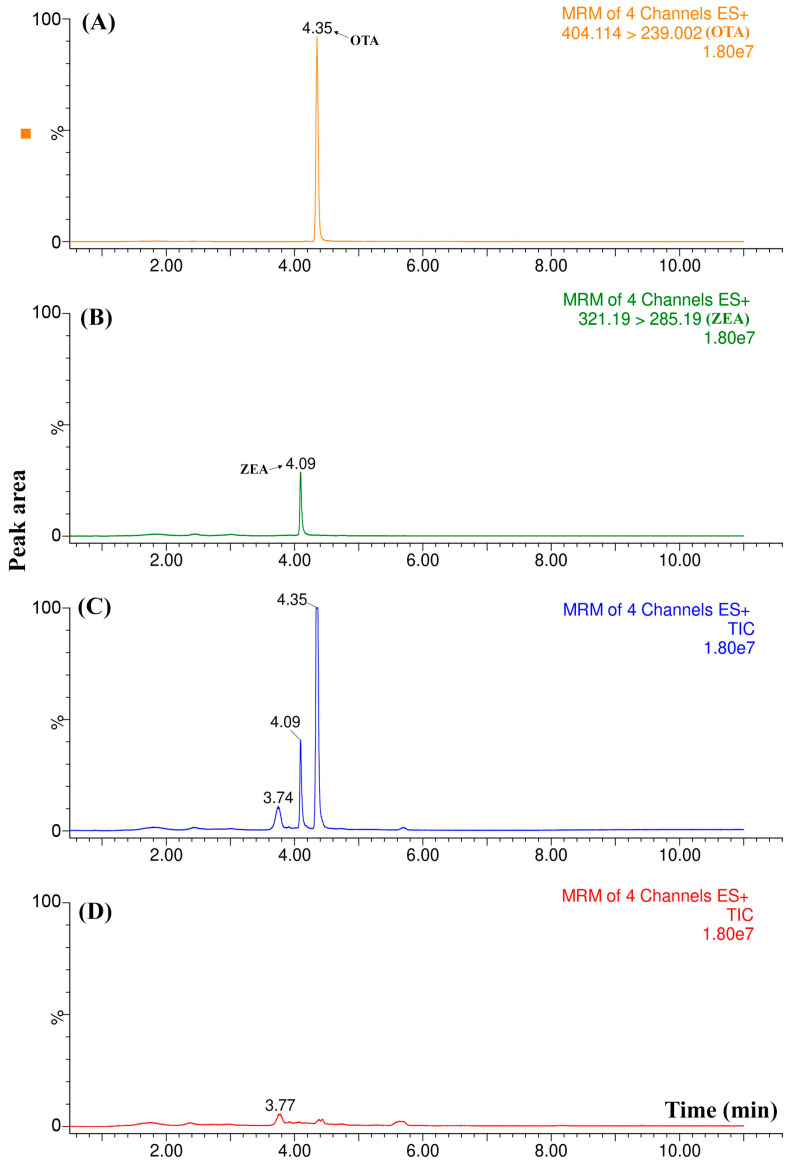
Representative chromatograms comparing a blank wine sample with one spiked at a concentration of at 15 µg L^−1^. (**A**) OTA ion transition; (**B**) ZEA ion transition; (**C**) total ion chromatogram of the spiked wine; and (**D**) total ion chromatogram of the blank wine sample.

The first test carried out consisted of injecting three spiked wine samples at 15 µg L^−1^ to verify the retention time (t_R_) reproducibility. Figure S5 (Supplementary Materials) shows that the t_R_ for OTA (4.35 min) and ZEA (4.09 min) perfectly matched, considering the samples were just filtered by a cellulose membrane (0.22 µm) before the analysis. These results suggest that the method could have eliminated most of the matrix interferents that could have affected the analysis. Although a signal can be identified at 3.7 min, this is not a problem, as it does not overlap with any signal at the mycotoxins’ retention time. In the sequence, blank wine samples were injected and compared with the chromatographic profiles previously acquired. The results obtained by employing the selected reaction monitoring (SRM) mode were compared by examining the total ion chromatogram (TIC) as well as each ion transition. As shown in Figure 4, there was no signal for OTA or ZEA in the blank sample (D), suggesting it was free of mycotoxin contamination. However, there is an interferent signal from the matrix that was observed at 3.74 min, related to a ZEA identification ion transition, which was not the most intense, and thus is not shown in Figure 4. Once no signal overlaps with those of OTA and ZEA are monitored, the analytical method can be considered selective.

In addition to the wine samples, coffee and almond liquors were analyzed. These samples were chosen due to the known possibility of the presence of mycotoxins, considering their raw material origin: almond and coffee. For this stage, apart from OTA and ZEA, four aflatoxins were also included in the method scope (AFB_1_, AFB_2_, AFG_1_, and AFG_2_). Figure S6 (Supplementary Materials) shows the total ion chromatogram (TIC) for the spiked almond liquor (A), spiked coffee liquor (B), and a spiked standard solution (C) containing the target analytes at 15 µg L^−1^. As can be seen, the automated analysis, based on setup 2, can also be used to analyze other complex matrices potentially related to mycotoxin contamination. This versatility is an excellent characteristic of this automated system 2, when used for more than one matrix and several different target mycotoxins. Another essential characteristic is their short run time analysis, reinforcing the high throughput of the automated methods. In the proposed method, just a simple filtration with a cellulose membrane was carried out before the unattended analysis. In this way, several samples could be processed daily (ca. 6 samples per hour), enhancing productivity, while the use of miniaturized approaches allow a significant economy of solvents and samples (injection volume of 1 µL) while generating less waste.

**Figure 5:**
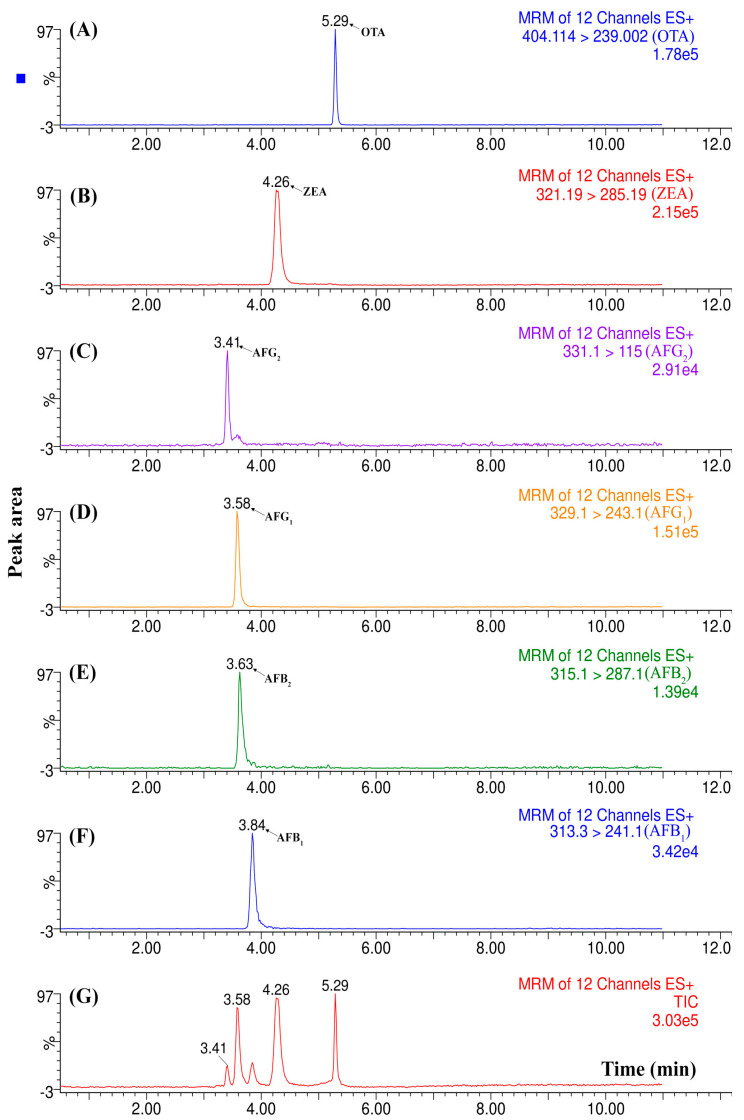
Total ion chromatogram (TIC) and selected reaction monitoring (SRM) of each mycotoxin analyzed in a spiked almond liquor sample. (**A**) OTA ion transition; (**B**) ZEA ion transition; (**C**,**D**) AFG_1_ and AFG_2_ ion transitions (Aflatoxin G_1_, Aflatoxin G_2_); (**E**,**F**) AFB_1_ and AFB_2_ (Aflatoxin B_1_, Aflatoxin B_2_) ion transitions; (**G**) TIC of the analyzed sample.

For the last example, Figure 5 depicts the results of almond liquor analyzed using the selected reaction monitoring (SRM) mode, highlighting the more intense ion transition for each mycotoxin monitored. It is noteworthy that the AFB2 chromatographic peak (3.63 min) did not appear in the TIC (G), because it was much less intense, and thus was suppressed by the others. However, when the ion transition was isolated (E) we can see it properly. Additionally, the good overall selectivity obtained when using in-tandem MS as a detection method can help our system 2 to enhance its capacity even more to detect multi-mycotoxin signals at low concentration levels. Similarly to setup 1, the LOQs were determined by calculating the concentration values at a signal-to-noise ratio of 10 (S/N 10:1) for each analyte. In this way, the reported LOQs were: wine (OTA = 110 ng L^−1^; ZEA = 380 ng L^−1^), almond liquor (OTA = 320 ng L^−1^; ZEA = 620 ng L^−1^; AFG_1_ = 350 ng L^−1^; AFG_2_ = 1110 ng L^−1^; AFB_1_ = 450 ng L^−1^; AFB_2_ = 1000 ng L^−1^), and coffee liquor (OTA = 350 ng L^−1^; ZEA = 780 ng L^−1^; AFG_1_ = 600 ng L^−1^; AFG_2_ = 540 ng L^−1^; AFB_1_ = 510 ng L^−1^; AFB_2_ = 1080 ng L^−1^).

#### 2.2.4. Method Overview

Despite some publications that report the use of automated or miniaturized methods for mycotoxin sample preparation, the field is still predominantly consists of non-automated and conventional approaches [15,21,22,23,25,29]. Additionally, from these publications, we can see the occurrence of sample preparation methods based on the association of automation and miniaturization is rare. The proposed systems 1 and 2 aim to link the best of them (automation and miniaturization) for such purposes.

In this section, centered on system 2, some of its main characteristics should be highlighted. Looking at the miniaturization benefits, it is unarguably economical, regarding the reagent consumption and waste generation of this method. Comparing to other recently published approaches [21,22,23], our system 2 works with approximately a hundred times less mobile phase, as well as requiring less than 4% of “real” samples than the referred works. Likewise, methods requiring mycotoxin sample preparation could demand preliminary steps [33,34], such as centrifugation, dilution, shaking, or filtration. It is noteworthy that in this work, due to the process automation, wine samples should be filtered immediately before injection, allowing the analysis of up to six samples per hour. Another critical advantage that could be reinforced is the simple process of synthesizing and using our extractive phases based on graphene. Some published works report the use of immunoaffinity sorbents for mycotoxin sample preparation, which in general are more complex sorbents to work with, as well as being more expensive to acquire [24,35]. Besides, the proposed extraction column might be utilized for more than 250 analyses without modifying its original sorptive capacity.

From the automation viewpoint, system 2 also exhibits some favorable characteristics, especially emphasizing its more straightforward operation mode, since only a 0.22 µm sample filtration must be done before the injection. Considering that after this action, all analysis steps are controlled by the instrument software, it brings three significant features: (i) the possibility of remote-operated analyses, (ii) increasing analysis throughput, and (iii) low manual handling, which decreases the potential occurrence of analytical errors. Furthermore, considering the miniaturization of the extraction part and the whole analytical technique (including capillary LC), system 2 provides a high chromatographic peak profile and excellent signal intensity. This configuration might be a promising alternative to developing greener analytical methods without sacrificing the quality of the analytical results, which is within the current trends of green chemistry.

Finally, a brief comparison between some analytical characteristics of systems 1 and 2 is shown in Table 2. Following this, some observations can be highlighted: considering the purpose of these two systems, both exhibit good analysis time, less than 20 min in total. Another aspect to consider is the relation between the LOQs and injection volume. As can be seen, system 1 reached inferior LOQs compared to system 2, due to the large injection volume available on it (50 µL). This characteristic suggests it as a more suitable configuration for dealing with the lower concentration levels investigated in common matrices such as domestic coffee, beer, and wine samples. Conversely, when the analysis of a more expensive matrix is desirable (e.g., imported beverages like those liquors herein analyzed), system 2 seems more adequate, since it can achieve satisfactory LOQs by using around fifty times lower sample volumes. Indeed, when high throughput analysis is the main goal, system 2 arises as a promising configuration, as it put together all benefits of an online sample preparation approach with a good efficiency of capillary liquid chromatography, even in reduced sample volumes. Assuredly, both configurations generate small volumes of waste, which was one of our main goals: proposing methods for mycotoxin analysis without compromise with the environmental principles of green chemistry.

## 3. Experimental

### 3.1. Standards and Reagents

Ochratoxin A analytical standard (OTA) 99.5% and Zearalenone (ZEA) 99.5% were both purchased from Fluka-Analytical (St. Louis, MO, USA). A standard mix of aflatoxins (99.5%) containing AfB_1_, AfB_2_, AfG_1_, and AfG_2_ solubilized in benzene:acetonitrile (98:2) were acquired from Merck (Darmstadt, Germany).

HPLC grade acetonitrile (ACN) methanol (MeOH), and formic acid (FA) 98% grade AR, were purchased from Tedia (Fairfield, OH, USA), and ultra-high purity (UHP) water was supplied from a Milli-Q system (Burlington, MA, USA).

### 3.2. Mycotoxin Standard Solutions

OTA and ZEA stock solutions of 150 mg L^−1^ were made in methanol and stored at −18 °C in an amber vial. The aflatoxin stock solution was prepared from a standard mix dilution to a concentration level of 0.5 mg L^−1^, this solution was used to prepare each work solution before chromatographic injections.

The exact concentration of the OTA stock solution was determined by UV–Vis spectrophotometry [11]. For this, 1.00 mL of stock solution was placed in a vial and dried with N_2_, and then dissolved in 1.00 mL of toluene: acetic acid (99:1, *v*/*v*). This solution was transferred to a 1.0 mL quartz cell, and the concentration was determined at λ_max_ = 333 nm. Working solutions, prepared and used as needed, were made in the mobile phase solvent from the stock solution of 30 mg L^−1^ of OTA in MeOH.

### 3.3. Extraction Column Packing Procedure

First, a Haskel DSHF-122 hydropneumatic pump from Haskel (Burbank, CA, USA) was employed to pack both extraction columns utilized in setups 1 and 2. Although both extraction dispositive was packed by the same instrumentation, some differences in the operating procedure and the sorbent phase are stated next.

Setup 1 employed as a sorbent the GO anchored on aminopropyl silica particles and functionalized with octadecylsilane and trimethylsilane (SiGOC18ecap). The synthesis and characterization of this material are described by Mejía-Carmona et al. [32]. Approximately 20.0 mg of SiGOC18ecap was packed in a stainless steel capillary column (508 µm i.d. × 100 mm) by a slurry packing method using a solution of THF:IPA (1:6, *v*/*v*) as the slurry solvent to suspend the sorbent, and ultrapure water as the packing solvent.

The extraction column of setup 2 was produced by packing approximately 10.0 mg of sorbent (GO-Sil) into stainless steel tubing (254 µm i.d. × 100 mm) using the same slurry and packing solvents. The synthesis and characterization of the GO-Sil are described by Maciel et al. [36]. Figure S7 (Supplementary Materials) shows a picture of the hardware required to assemble the miniaturized extraction columns utilized in this work.

### 3.4. Setup 1: Assembly and General Characteristics

For this setup, an Acquity UPLC liquid chromatography from Waters (Milford, MA, USA) supplied with a binary solvent manager and autosampler was used, coupled to a XEVO TQ Mass Spectrometer with an electrospray ionization (ESI) interface, all from Waters (Milford, MA, USA). Chromatographic separation was performed in an analytical column Poroshell 120 SB-C8 (2.1 mm d.i. × 100 mm; 2.7 µm d.p.), used under the following conditions: column temperature, 40 °C; injector temperature, 15 °C; injection volume, 50 µL in full-loop mode; flow rate 0.200 mL min^−1^, mobile phases A: H_2_O 0.1% formic acid, and B: ACN 0.1% formic acid, in stepwise elution gradient mode (see blue line in Figure 1). The injector needle was washed between each injection with an additional washing cycle.

ESI conditions: positive ionization mode; capillary voltage, 3.0 kV; cone voltage, 16 V; source temperature, 150 °C; desolvation temperature, 400 °C; solvation carrier gas N_2_, 800 L h^−1^; collision gas flow Ar, 0.15 mL min^−1^. Tandem MS/MS conditions were optimized by direct infusion of the OTA standard solutions at 100 µg L^−1^, data were acquired in selected reaction monitoring (SRM) mode by MassLynx 4.1 software from Waters (Milford, MA, USA), and dwell time, 0.62 s. Selected quantification OTA transition was *m*/*z* 404 → *m*/*z* 239 (cone voltage, 24 V; collision energy, 26 eV), and confirmation transition was *m*/*z* 404 → *m*/*z* 221 (cone voltage, 24 V; collision energy, 36 eV).

**Figure 6:**
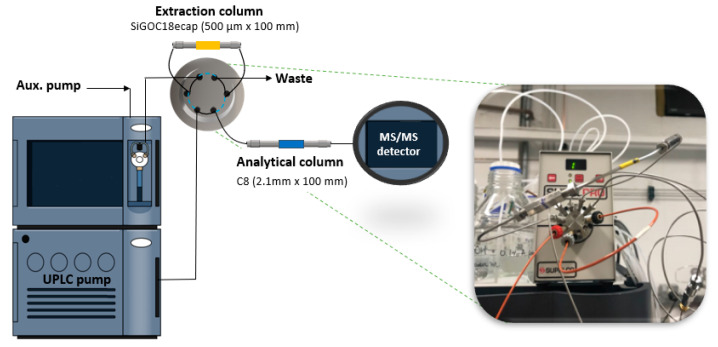
Representative scheme of setup 1, obtained by a coupling of the extraction microcolumn to a UPLC–MS/MS system, in flow-through mode. A six-port valve is shown in the loading (black trace) and elution (blue trace, back-flush mode) positions. In the inset, a photograph depicts the valve system.

For assembling the multidimensional set up (Figure 6), the microextraction column was connected to the UPLC–MS/MS system using an external Supelpro^®^ six-port valve (operated by the chromatograph software in two positions, 1: loading and 2: elution), and a Shimadzu LC-20AT auxiliary pump. The sample loading/extraction was made in the flow-through mode and the elution in the back-flush mode. In the loading position, 50 µL (full-loop mode) of the sample was injected and carried to the extraction microcolumn by the loading phase (H_2_O:ACN (85:15, *v*/*v*) acidified with 0.1% formic acid at 0.150 mL min^−1^ flow rate) using the auxiliary pump, which was connected to the auto-injector. The sample was flushed for 4 min through the extraction microcolumn; the target compounds were retained, while matrix interferents were eliminated through the waste. Subsequently, the valve was switched to the elution position, and the UPLC mobile phase passed through the extraction microcolumn in a back-flush flow at 0.200 mL min^−1^, over 16.5 min in a step gradient. The first solvent gradient (H_2_O:ACN (40:60, *v*/*v*) 0.1% formic acid) transferred the OTA to the analytical column to be separated and quantified by the MS/MS detector. The second gradient (H_2_O:ACN (5:95, *v*/*v*) 0.1% formic acid) did the clean-up of both columns (extraction and analytical). Finally, after 5 min of conditioning, system 1 was ready for other injections. The scheme of the time used to operate the valves is depicted in Figure 1 (see boxes).

### 3.5. Setup 2: Assembly and General Characteristics

**Figure 7:**
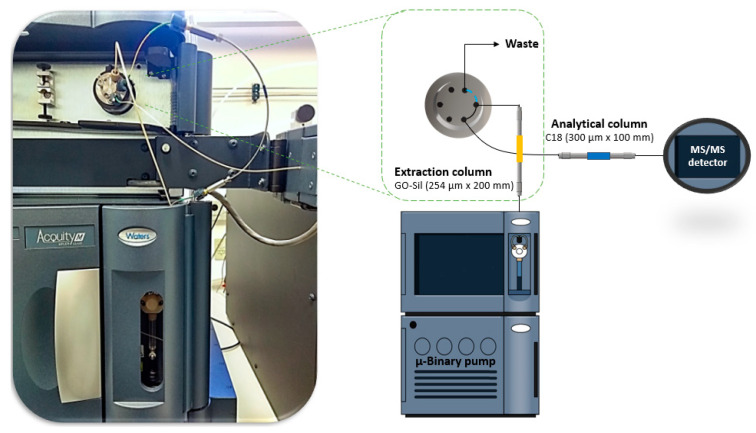
Representative scheme of the system 2 configuration. An actual photograph of the switching valve connections and the capillary extraction column is depicted in the left, while a drawing of the whole system is displayed on the right side of the figure.

In this case, setup 2 was composed of an Acquity M-Class for liquid chromatography supplied with a µ-Binary solvent manager (pumping system), a sample manager (injector), and a trap valve manager (switching valve) coupled to a Xevo TQ S Micro (MS/MS), the whole equipment setup was acquired from Waters (Milford, MA, USA). Figure 7 shows a representative scheme of the assembled system on the right, while an actual photograph of the capillary extraction column connected to the trap valve manager is enlarged on the left.

The extraction column used was a lab-packed GO-Sil with capillary dimensions (254 µm × 200 mm; 50 µm d.p.). Likewise, the chromatographic separation was performed using a C18 THS capillary column (300 µm × 100 mm; 1.7 µm d.p.), purchased from Waters (Milford, MA, USA), operating at a temperature of 35 °C with the mobile phase composed of ultrapure water (A) and acetonitrile (B), both 0.1% acidified with formic acid, under a flow rate of 10 µL min^−1^. The in-tandem MS detection was performed using the selected reaction monitoring mode (SRM) responsible for identifying the specific ion transitions for OTA, ZEA, and the four aflatoxins. The monitored transitions were selected by the direct infusion of a standard solution of each analyte (50 µg L^−1^) using the assisting software IntelliStart 4.2, from Waters (Milford, MA, USA). The analyte transitions and their main detector parameters are presented in Table S1 (Supplementary Materials). Other important MS/MS detection parameters included: positive electrospray ionization; capillary voltage, 3.5 kV; source temperature, 150 °C; desolvation temperature, 350 °C; desolvation gas N_2_, 900 L h^−1^; and collision gas Argon (Ar).

After system 2’s short description, an explanation about the working steps is presented. The mycotoxin automated analysis was performed in two different steps: (i) automated extraction and (ii) capillary LC–MS/MS analysis. The trap valve manager was responsible for controlling the mobile phase flow direction, alternating between loading and elution positions. Therefore, in the first step after the 1 µL sample injection by the equipment, only the GO-Sil extraction column received the mobile phase flow at 25 µL min^−1^, composed of 0.2% FA acidified H_2_O:ACN (78:22, *v*/*v*). During this, the µ-binary solvent manager pushed the sample from the loop (1 µL) into the extraction column, over 1 min, to load the target analytes in the extractive phase while most of the matrix interferents were eliminated through the waste. Subsequently, the valve was switched to the elution position, and then the µ-binary solvent manager pumped a flow of 10 µL min^−1^ into the extraction column connected in-line with the analytical column to separate and detect the target compounds. In this case, the mobile phase had the same composition as the extraction step, but an elution gradient was employed (Figure S3, Supplementary Materials). Finally, after ten minutes of analysis, system 2 was ready for the next injection.

### 3.6. Sample Preparation

For system 1, wine and beer samples were degassed in ultrasound for 5 min, then spiked with OTA at the specified concentration and filtered through a 0.22 μm cellulose membrane. Instant coffee samples were ground until a fine powder was obtained. One gram of the sample was dissolved with 100 mL of boiling water, shaken (1000 rpm, 10 min), and centrifuged (14,500 rpm, 10 min). An aliquot of the supernatant was spiked with OTA, at the specified concentration, and filtered through a 0.22 µm cellulose membrane before the analysis.

For system 2, the samples of wine, and almond and coffee liquors were just spiked with mycotoxins (OTA, ZEA, and AFAs) at the specified concentration and filtered through a 0.22 μm cellulose membrane.

## 4. Conclusions

This work presents two different setups to analyze residues of ochratoxin A, zearalenone, and four aflatoxins (B_1_, B_2_, G_1_, and G_2_) in complex matrixes. The focus was on the development and evaluation of greener analytical methods aimed to correlate the benefit of miniaturization and automatization for the field of mycotoxin analysis. In this context, both analytical systems (1 and 2) consisted of an automated sample preparation method followed by liquid chromatography coupled to in-tandem mass spectrometry. In short, system 1 consisted of a lab-made microextraction column packed with a new SiGOC18ecap sorbent phase, which presented excellent extraction performance, resulting in good analytical responses up to a concentration of 2 µg L^−1^. In this case, a fully automated sample preparation of beer, wine, and instant coffee was performed before OTA identification. Another interesting point here is the cheap extraction hardware employed, which costs under US$50 and can perform more than 250 injections before losing its original working condition. Additionally, due to the microextraction column dimension, only 20 mg of the sorbent phase was packed into it, while the sample required for the method was only 50 µL, being much less than is used for conventional sample preparation techniques based on QuEChERS (Quick, Easy, Cheap, Rugged, and Safe) and SPE, among others [23,29,33,37]. Similarly, the method proposed in system 2 was primarily based on the benefits obtained by allying miniaturization and automation in one configuration. For this reason, a new assembly employing a 254 µm i.d. extraction capillary column packed with a graphene-based sorbent (GO-Sil), was employed in the fully automated extraction of several mycotoxins in wine samples, and almond and coffee liquors. The miniaturized sample preparation was further coupled to capillary LC–MS/MS to emphasize, even more, the impact of miniaturization on the economy of chemicals and samples. The estimated consumption of reagents and waste generation of system 2 is shown to be 100 times lower than in recently published works [21,22,23] while it also demands not more than 4% of the amount of sample commonly required [33,34]. Like in system 1, the in-lab made extractive phase showed good behavior, performing more than 250 injections. When looking at the robustness of the two lab-packed extraction columns, these methods showed a profit perspective, since commercially available SPE-based extraction devices are usually disposable. Another attractive characteristic was the absence of widely employed additional steps, such as centrifugation, dilution, and the shaking of samples before the extraction step. Due to this, the total analysis time was approximately ten minutes, and manual handling was kept to a minimum in both methods. By comparing them, system 1 seems to be more suited to the analysis of mycotoxins in common matrices (such as coffee and wine) when a larger volume of samples is available and lower limits of quantification are required. Contrariwise, when higher throughput is the main goal, and a lower reagent cost is desirable, system 2 might be a good configuration. As a drawback, to assemble the configurations used in these setups, a switching valve and an auxiliary pump must at least be present, beside the analytical equipment. However, the authors thought that the benefits rising from miniaturization and automation could make such a kind of instrumentation cheaper in the long run. For all these reasons, this manuscript aimed to present and evaluate through some analytical experiments on the performance of two fully automated and miniaturized different methods for mycotoxin analysis. From our point of view, this system exhibits excellent performance and represents a suitable alternative to achieve high-throughput methods that are environmentally friendly at the same time.

## Figures and Tables

**Table 1:** Evaluation of the OTA *m*/*z* 404.1 → *m*/*z* 238.9 ion transition response as a function of the analyzed matrix.

Matrix	Spiked OTA (μg L^−1^)	S/N Ratio	LOQ (ng L^−1^)
Instant coffee	2.0	57.02	351
Malt beer	2.0	271.73	74
Lager beer	2.0	230.35	86
Red wine	2.0	294.72	68

**Table 2:** Comparison between the main analytical characteristics of systems 1 and 2.

Parameter	System 1	System 2
Injected sample volume (µL)	50	1
Time per analysis (min)	19	10
Extraction flow rate (µL min^−1^)	150	25
Analysis flow rate (µL min^−1^)	200	10
Waste generated per analysis (mL)	6.65	0.12
LOQs (ng L^−1^) ^a^	68–86	110–1110

^a^ range considering all analytes.

## Supplementary Materials

The following are available online: Figure S1: Chromatograms obtained in SRM mode of the evaluation of the loading time from 1 to 6 min of a standard solution of OTA at 20 µg L^−1^ at 0.100 mL min^−1^ flow rate of H_2_O:ACN (78:22, *v*/*v*) acidified 0.1% formic acid; Figure S2: Wasting fractions collected during the online extraction step by each minute from 1 to 4 min (during the sample loading time, 4 min) of (A) wine and (B) instant coffee samples, both spiked at 20 µg L^−1^ with OTA; Figure S3: Step elution gradient employed in the multi-mycotoxin analysis by multidimensional capillary LC–MS/MS. Mobile phases → A: H_2_O: 0.1% formic acid and B: ACN: 0.1% formic acid (blue line). The box below the gradient indicates the configuration of the switching valve (*); Figure S4 Performance (peak area vs. analytes) of the analytical parameters considered in the extraction method enhancement, carried out by univariate experiments (*n* = 3). (A) Loading time; and (B) loading flow; Figure S5: Total ion chromatograms of three spiked wine samples (15 µg L^−1^) to illustrate the retention time reproducibility and chromatographic behavior profile; Figure S6: Representative chromatograms showing: (A) almond liquor, (B) coffee liquor, and (C) standard solution. All of them were spiked at a concentration of at 15 µg L^−1^; Figure S7: Typical microcolumn hardware used as an extraction device in both systems; Table S1: Analytes’ precursor and product-ion and its main detection parameters utilized in the selected reaction monitoring MS mode.

## Abbreviations

ACN	acetonitrile
AFAs	aflatoxins
AFB_1_	aflatoxin B_1_
AFB_2_	aflatoxin B_2_
AFG_1_	aflatoxin G_1_
AFG_2_	aflatoxin G_2_
ESI	electrospray ionization
FA	formic acid
GO-Sil	graphene oxide supported on amino silica
HPLC	high-performance liquid chromatography
IAC	immunoaffinity columns
i.d.	inner diameter
LC	liquid chromatography
LLE	liquid–liquid extraction
MeOH	methanol
MS	mass spectrometry
MS/MS	tandem mass spectrometry
OTA	ochratoxin A
SiGOC18ecap	graphene oxide anchored to aminopropyl silica particles and functionalized with octadecylsilane and trimethyl silane
SRM	selected reaction monitoring
SPE	solid-phase extraction
SPME	solid-phase microextraction
UPLC	ultra-performance liquid chromatography
TFC	turbulent flow chromatography
ZEA	zearalenone
