# Supplementary material for: Evaluation of Two Fully Automated Setups for Mycotoxin Analysis Based on Online Extraction-Liquid Chromatography–Tandem Mass Spectrometry
Source: Molecules. 2020 Jun 15;25(12):2756. doi: 10.3390/molecules25122756 (PMC7356336; doi:10.3390/molecules25122756)
Supplement: Supplementary file 1 [file molecules-25-02756-s001.pdf]

molecules

Supplementary Material

Evaluation of Two Fully Automated Setups for Mycotoxin Analysis Based on Online Extraction–Liquid Chromatography–Tandem Mass Spectrometry

Edvaldo Vasconcelos Soares Maciel, Karen Mejía-Carmona and Fernando Mauro Lanças *

São Carlos Institute of Chemistry, University of São Paulo, São Carlos 13560-970, SP, Brazil; daltoniqsc@gmail.com (E.V.S.M.); ksmejiac@gmail.com (K.M.-C.)
* Correspondence: flancas@iqsc.usp.br; Tel.: +55-163373-9984

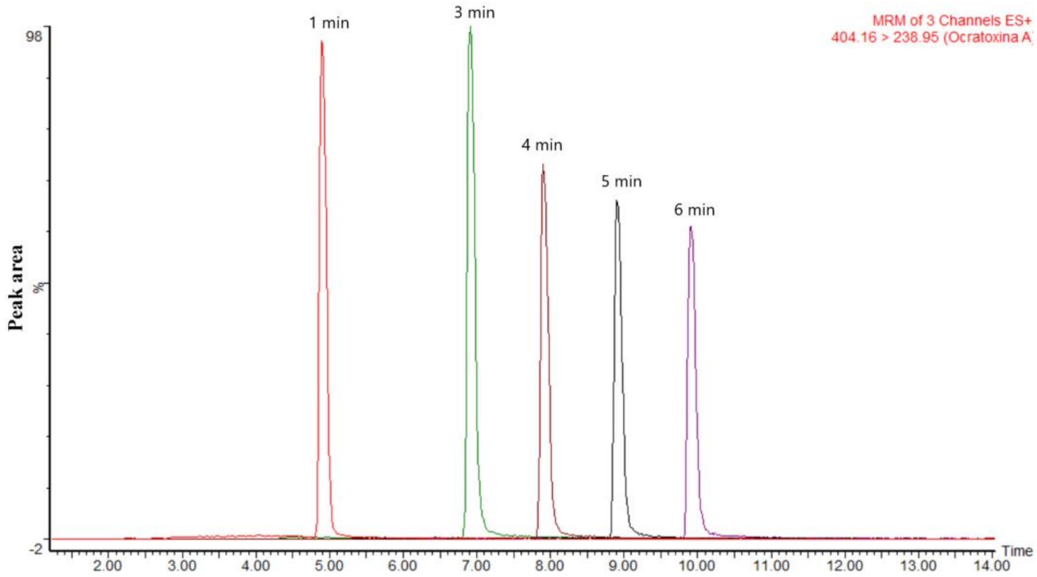

Figure S1. Representative chromatogram (SRM mode) obtained during the evaluation of the influence of the loading time (from 1 to 6 min) in the online automated LC-MS/MS system 1 employing a standard solution containing OTA at a concentration of 20 µg L^{-1}. The mobile phase used of H_2O: ACN (78:22, v/v) acidified with 0.1% formic acid, at a flow rate of 0.100 mL min^{-1}.

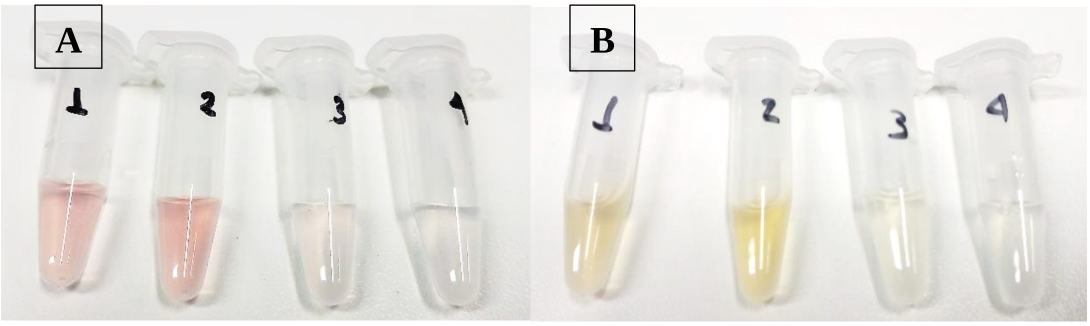

Figure S2. illustrates the four waste fractions collected by the discharge valve from 1 to 4 minutes for wine (A) and instant coffee (B). For both matrices, it is observed that in fractions 1, 2, and 3, most of the sample staining (polar fraction), were still being eliminated through the waste valve. On the contrary, Fraction 4 is translucent at this loading time (4 min), showing that the chosen parameters are adequate for providing the sample clean-up while retaining most of the OTA in the microextraction column.

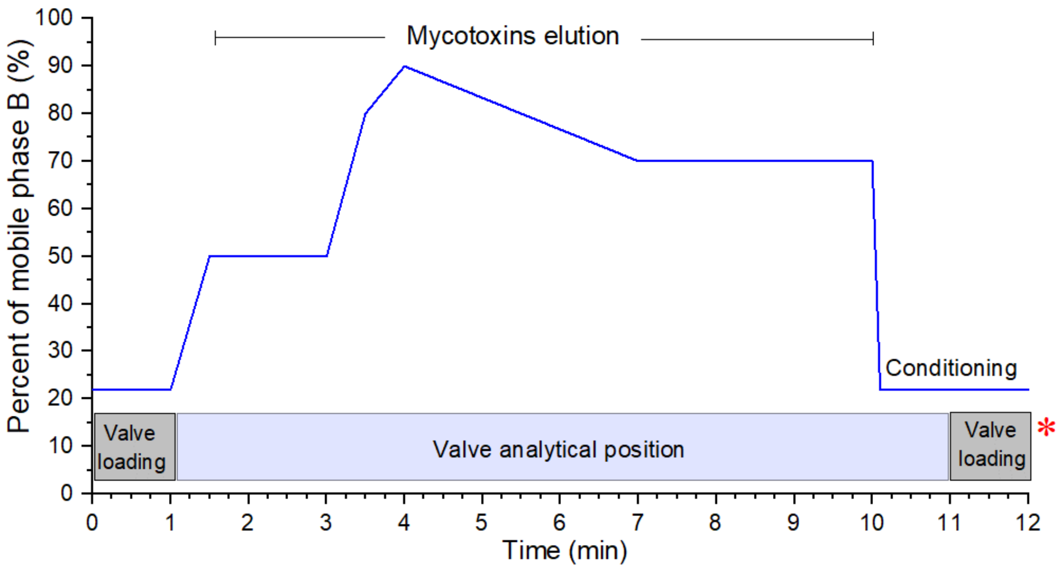

Figure S3. – Illustrative representation of the step elution gradient employed for the multi-mycotoxin analysis by multidimensional capillaryLC-MS/MS (system 2). Employed mobile phases → A: H_2O: 0,1% formic acid and B: ACN: 0,1% formic acid (blue line). Obs: The box below the gradient indicates the configuration of the switching valve (*).

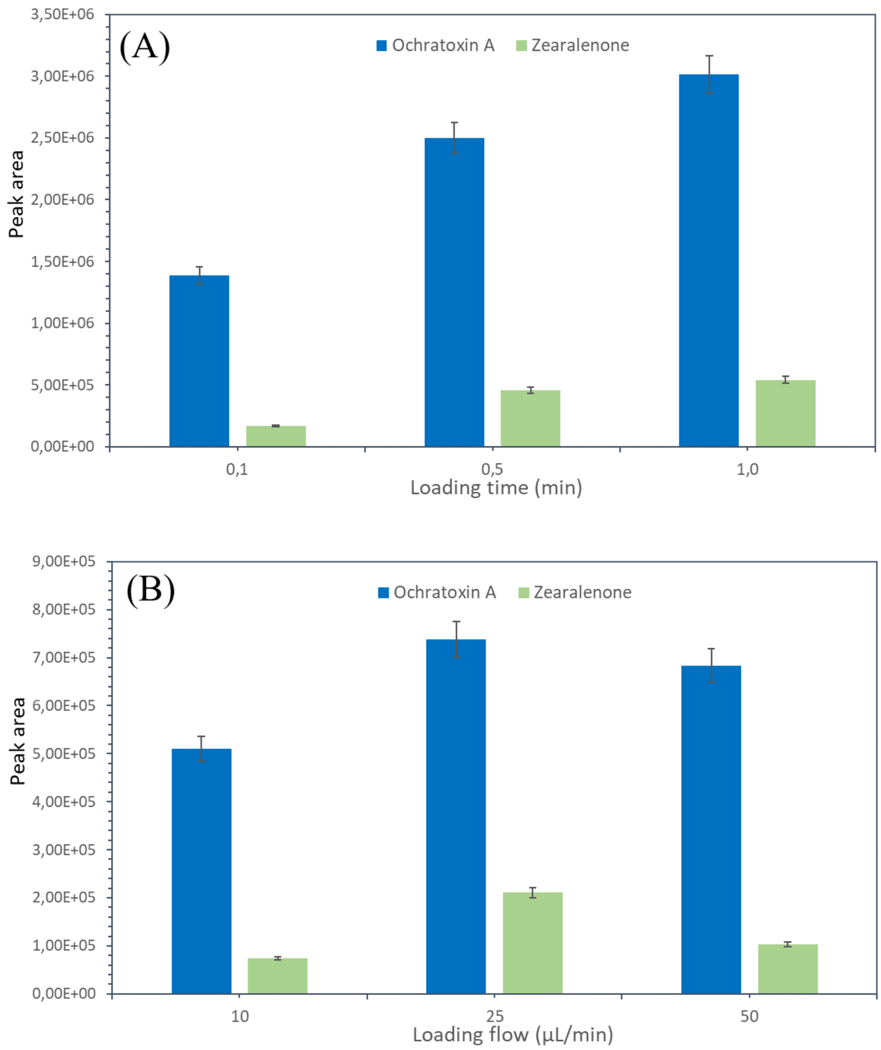

Figure S4. – Achieved performance (peak area vs. analytes) of the analytical parameters considered in the extraction method enhancement step for system 2. The tests were carried out by univariate experiments (n=3). (A) Loading time and (B) Loading Flow. This result is discussed in detail in section 2.2.4 in the main manuscript.

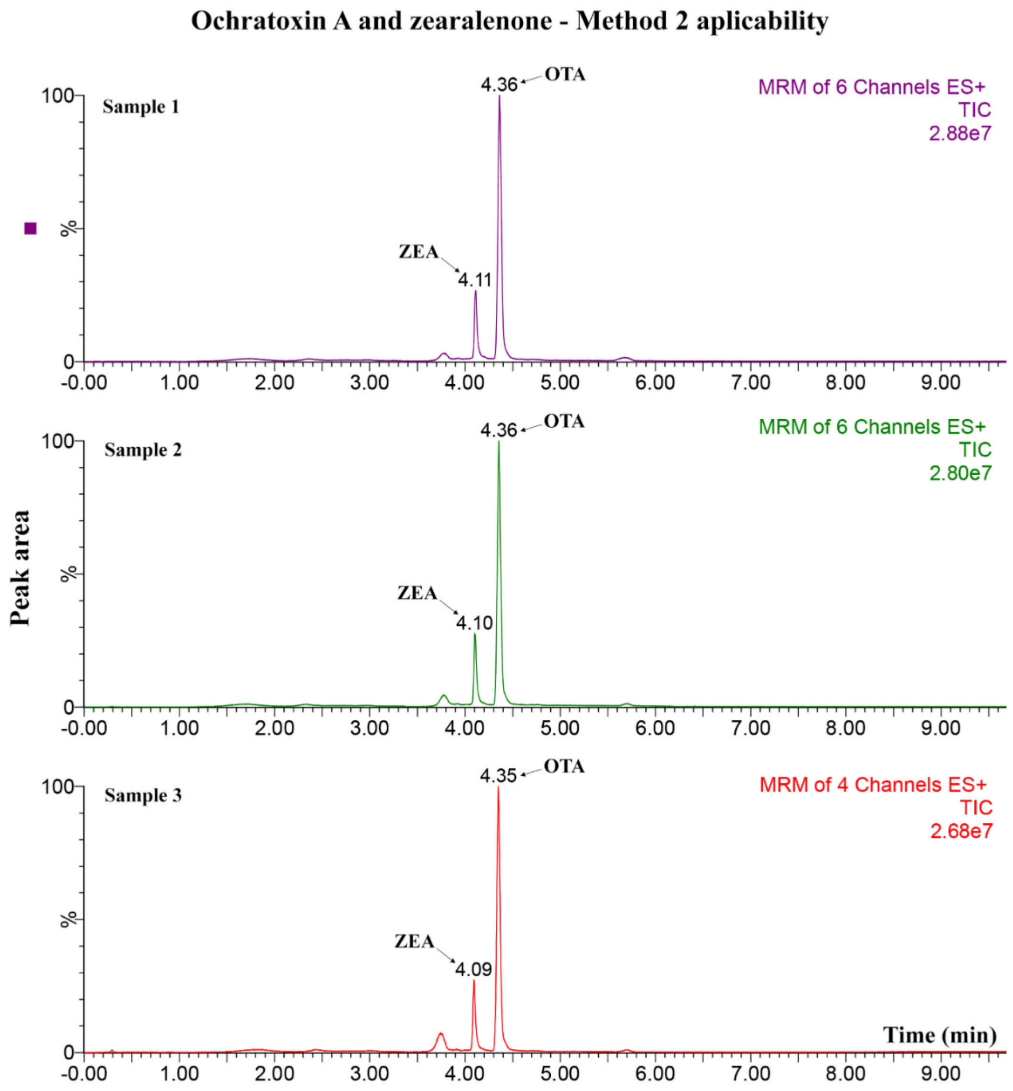

Figure S5. - Total ion chromatograms (TICs) of three spiked wine samples at a concentration of 15 μg L^{-1}, illustrating the retention time reproducibility and chromatographic profile of the target analytes. This evaluation was carried out to testify that the chromatographic separation method developed discussed in section 2.2.1 was adequate to be used during the other analytical steps.

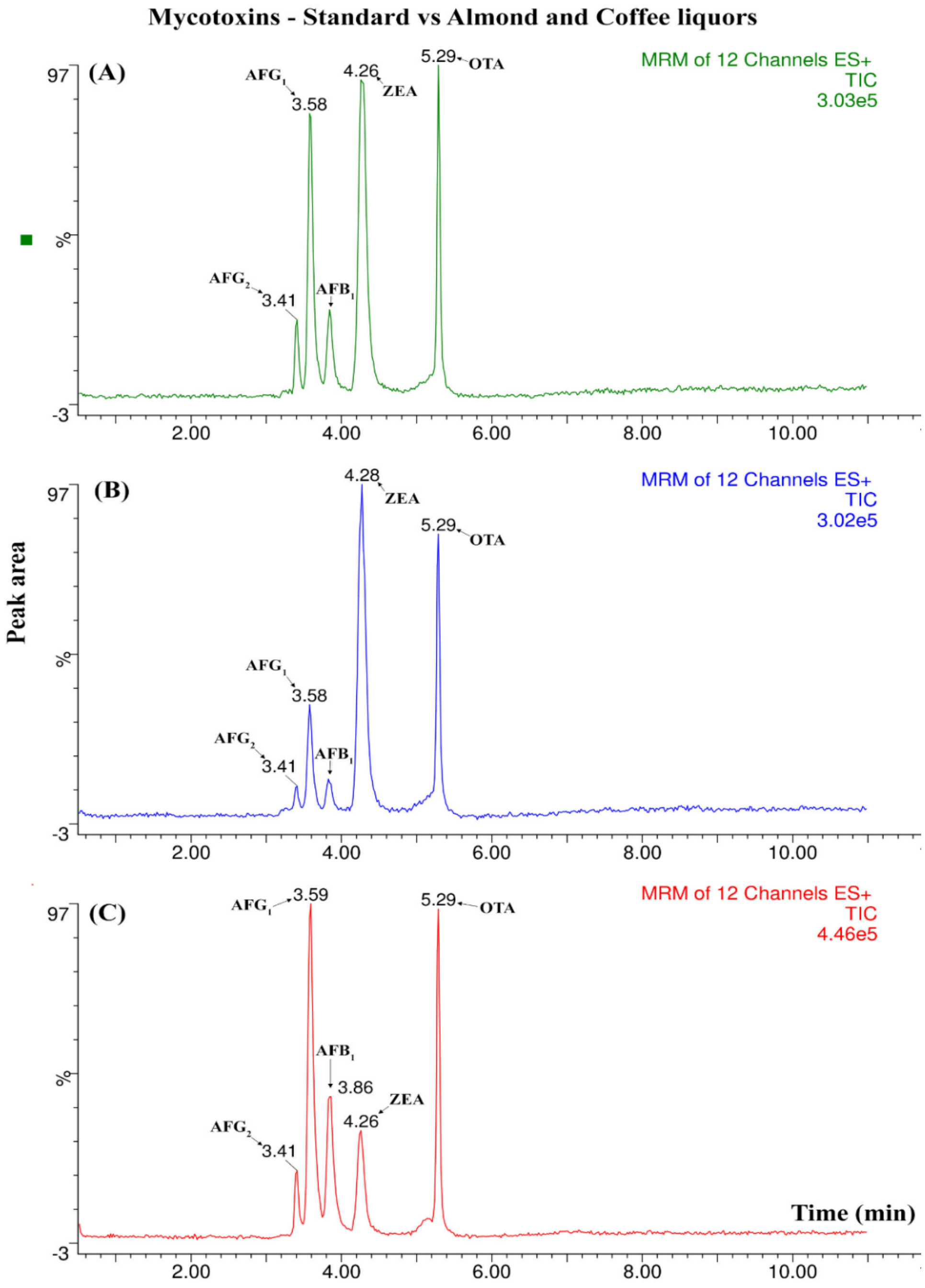

Figure S6. - Representative chromatograms corresponding to the online extraction-LC-MS/MS analysis of (A) almond liquor, (B) coffee liquor, and (C) standard solution. All samples were previously spiked with the analytes at a concentration of at 15 µg L^{-1}. As you can see, there are only small differences between the chromatographic profile obtained in (A), (B) and (C). This suggests that system 2 was able to eliminate most matrix interferents from almond and coffee liquors once they possess similar chromatographic behavior of (C)), standard solution).

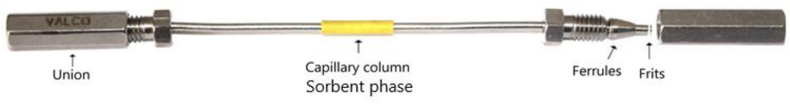

Figure S7. – Typical extraction microcolumn hardware (contains the sorbent inside), used as the extraction device in systems 1 and 2 described in this work.

Table S1. Precursor and product ions and its main detection parameters utilized in the MS/MS selected reaction monitoring (SRM) mode.

Analyte	Precursor ion (*m/z*)	Product ion (*m/z*)	Cone voltage (V)	Collision energy (V)
**AFB_1**	313	241	30	40
		285	30	20
**AFB_2**	315	259	30	28
		287	30	24
**AFG_1**	329	243	30	28
		311	30	20
**AFG_2**	331	115	30	80
		313	30	24
**OTA**	404	101	50	70
		239	50	24
**ZEA**	321	285	26	38
		67	26	30
